# Species History Masks the Effects of Human-Induced Range Loss – Unexpected Genetic Diversity in the Endangered Giant Mayfly *Palingenia longicauda*

**DOI:** 10.1371/journal.pone.0031872

**Published:** 2012-03-08

**Authors:** Miklós Bálint, Kristóf Málnás, Carsten Nowak, Jutta Geismar, Éva Váncsa, László Polyák, Szabolcs Lengyel, Peter Haase

**Affiliations:** 1 Biodiversity and Climate Research Centre, Frankfurt am Main, Germany; 2 Molecular Biology Center, Babeş-Bolyai University, Cluj, Romania; 3 BioAqua Pro Kft., Debrecen, Hungary; 4 Department of Ecology, University of Debrecen, Debrecen, Hungary; 5 Department of River Ecology and Conservation, Senckenberg Research Institute and Natural History Museum, Frankfurt am Main, Germany; 6 Department of Taxonomy and Ecology, Babeş-Bolyai University, Cluj, Romania; University of Illinois at Urbana-Champaign, United States of America

## Abstract

Freshwater biodiversity has declined dramatically in Europe in recent decades. Because of massive habitat pollution and morphological degradation of water bodies, many once widespread species persist in small fractions of their original range. These range contractions are generally believed to be accompanied by loss of intraspecific genetic diversity, due to the reduction of effective population sizes and the extinction of regional genetic lineages. We aimed to assess the loss of genetic diversity and its significance for future potential reintroduction of the long-tailed mayfly *Palingenia longicauda* (Olivier), which experienced approximately 98% range loss during the past century. Analysis of 936 bp of mitochondrial DNA of 245 extant specimens across the current range revealed a surprisingly large number of haplotypes (87), and a high level of haplotype diversity (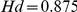). In contrast, historic specimens (6) from the lost range (Rhine catchment) were not differentiated from the extant Rába population (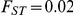, 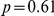), despite considerable geographic distance separating the two rivers. These observations can be explained by an overlap of the current with the historic (Pleistocene) refugia of the species. Most likely, the massive recent range loss mainly affected the range which was occupied by rapid post-glacial dispersal. We conclude that massive range losses do not necessarily coincide with genetic impoverishment and that a species' history must be considered when estimating loss of genetic diversity. The assessment of spatial genetic structures and prior phylogeographic information seems essential to conserve once widespread species.

## Introduction

Freshwater biodiversity is declining much faster than marine or terrestrial biodiversity [1]. Morphological degradation and pollution of water and sediments are the major drivers of biodiversity loss in stream systems around the world [2]. In central Europe, industrialization, combined with active channelization of large and small bodies of running waters, has led to a dramatic reduction in species diversity of fish, aquatic invertebrates, and other taxa [3]. While species extinctions have rarely been documented, numerous previously widespread species now only persist as small relict populations in refugia which were not subjected to severe habitat alterations. It is expected that these massive range losses also have led to considerable losses of genetic diversity [4]–[6], including highly differentiated evolutionary lineages, which can be referred to as cryptic species, or evolutionary significant units (ESU) [7], [8]. Local genetic variants and ESUs might harbor unique evolutionary potential, and provide the source of adaptation to future environmental change [9], [10]. These concerns are particularly relevant in freshwater systems, because several species show high levels of differentiation within their ranges, especially along the main catchments [11]–[13], and for species with a limited potential for overland dispersal [14]–[18]. Although existing works suggest that range losses should parallel the loss of genetic diversity [4]–[6], there is still little empirical information on the coupling of these processes, especially in freshwater species.

**Figure 1:**
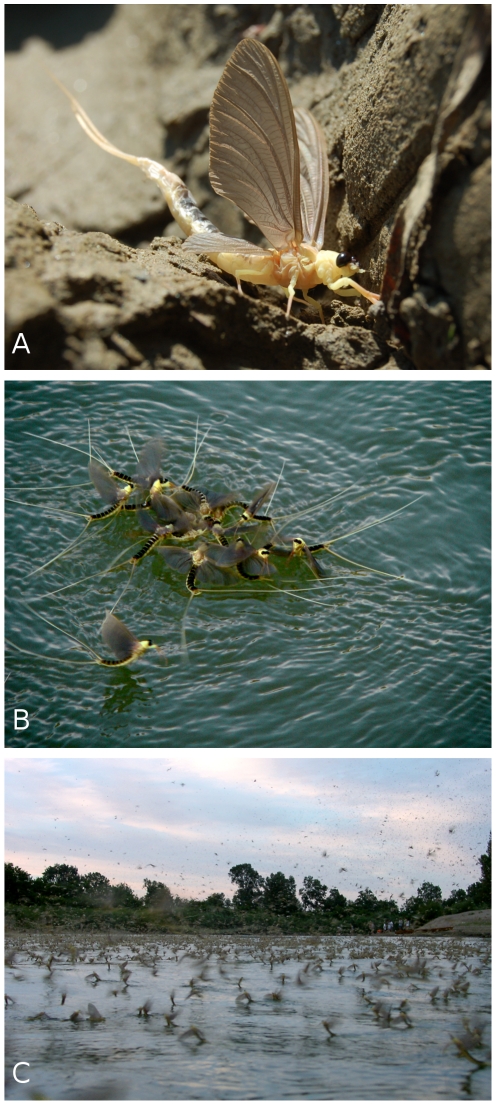
Long-tailed mayfly – *Palingenia longicauda*. A – freshly emerged Long-tailed mayfly (photo: A. Móra); B – males surrounding a female in a characteristic flower-like structure (“tiszavirág”) (photo: A. Orosz); C – the synchronized sunset swarming of millions of imagines (photo: L. Polyák).

**Figure 2:**
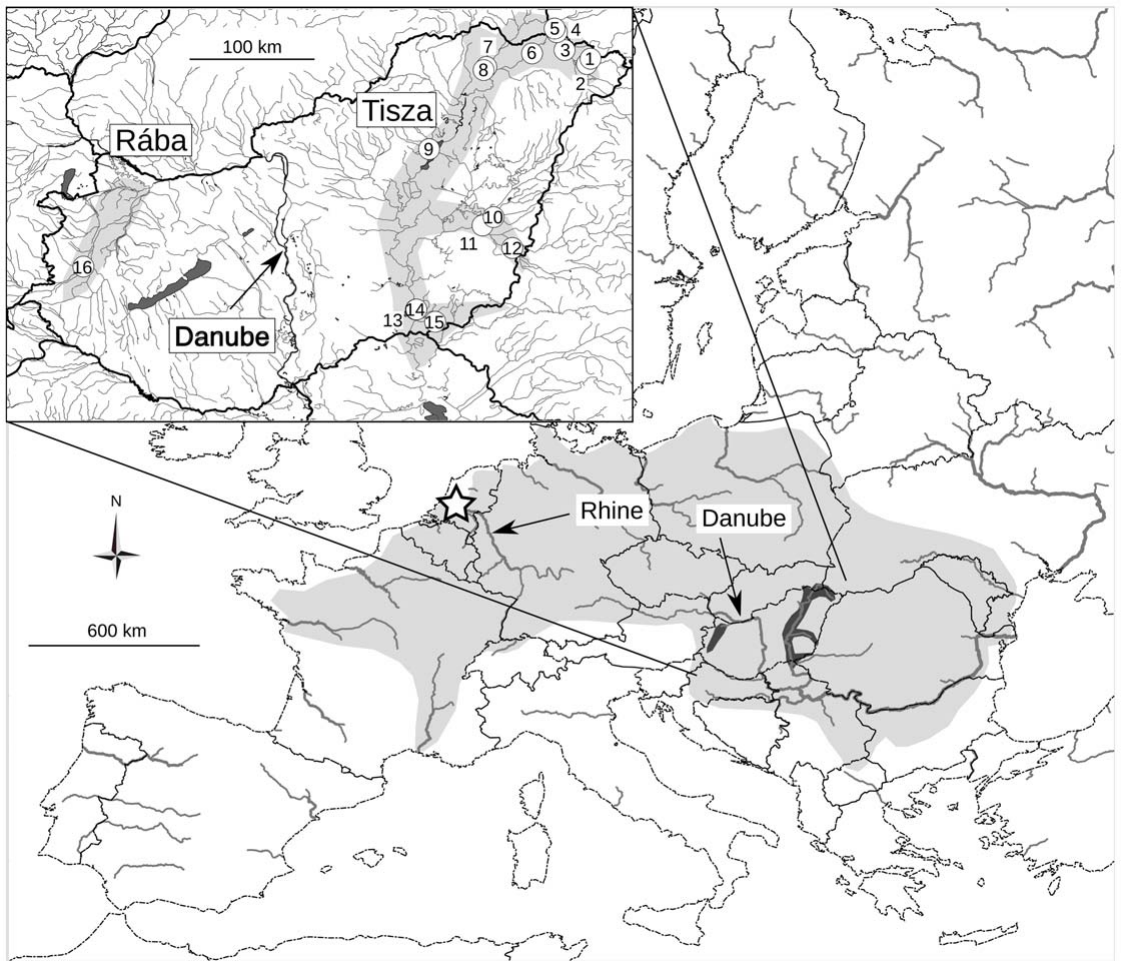
Former (light) and present (dark) distribution of *P. longicauda*. Approximate collecting locality of the historic Rhine specimens is marked with a star. Subset: collection sites of extant specimens, numbered according to Table S1. The former distribution range of the species was reconstructed after [19], [20].

In this context we chose the mayfly *Palingenia longicauda* (Ephemeroptera: Palingeniidae, Figure 1) to study the effects of severe range contraction on genetic diversity. This lowland riverine species used to be widespread and well-known from medium-large rivers in Europe, but today persists on about 2% of its former range (Figure 2) [19], [20]. Its dramatic decline coincided with the rapid, intensive hydromorphological alteration and pollution of European rivers and peaked around 1930 [20]. The species became extinct from the Loire (France) in 1922, from the Rhine (Germany) in 1952 and from the Danube (Serbia and Bulgaria) in 1973 [19]. *Palingenia longicauda* is presently restricted to the Tisza and the lower ranges of the river's tributaries in Hungary. It was rediscovered in the Rába river after 40 years of absence of reports [21]. The rapid and almost complete extinction of *P. longicauda* resulted in its listing as one of the few aquatic insects in Appendix II of the Convention on the Conservation of European Wildlife and Natural Habitats (Bern Convention) [22]–[24]. Current conservation attempts include protecting the species in its remaining native habitats in Hungary, as well as repopulating a Rhine tributary in Germany with Tisza source populations [25]. *Palingenia longicauda* has become a symbol of freshwater conservation efforts in Central Europe, especially in Hungary. This is due to its impressive body size (up to 10 cm) and reproductive behaviour, the spectacular synchronized swarming of millions of individuals (Figure 1A–C) [26].

We hypothesized that i) large-scale range loss will lead to high levels of range-wide genetic impoverishment in a once widespread aquatic species, ii) the rediscovery of this species in formerly lost ranges is due to recent range expansion, raising the hope for its re-establishment across Central Europe. We analyzed genetic diversity within the known refugial populations of *P. longicauda*. We compared the present genetic patterns with the haplotype diversity of historic museum specimens collected in the Rhine catchment, where the species is considered extinct. In addition, we compared the genetic structures in the known relict habitats (Tisza catchment) with a recently detected population from the Rába river in Hungary.

## Results

We obtained a 936-bp long sequence matrix for 245 extant specimens after combining mitochondrial COI (472 bp) and 16S (464 bp) sequences. There were no gaps in the 16S alignment. We successfully amplified a short fragment (196 bp) of the mtCOI gene in 24 museum specimens (Table S2). This was combined with mtCOI sequences of extant specimens into a 196-bp long matrix. All nucleotide sequences were deposited in European Nucleotide Archive (accession numbers, mtCOI: HE650151 – HE650419; 16S: HE650420 – HE650664). We were not able to amplify either the mtCOI or the 16S fragment for 17 extant specimens, and the short mtCOI fragment for 13 museum specimens (Table S2). Amplification success of the museum material varied with the place where the material was deposited. Many factors may have caused the repeated PCR failures, like field preservation inappropriate for DNA preservation (formalin, low concentration ethanol), “softening” dry specimens for further maceration, different aspects of storage conditions, etc. We cannot pinpoint a single cause which prevented PCR amplifications from the apparently well-preserved museum specimens.

**Figure 3:**
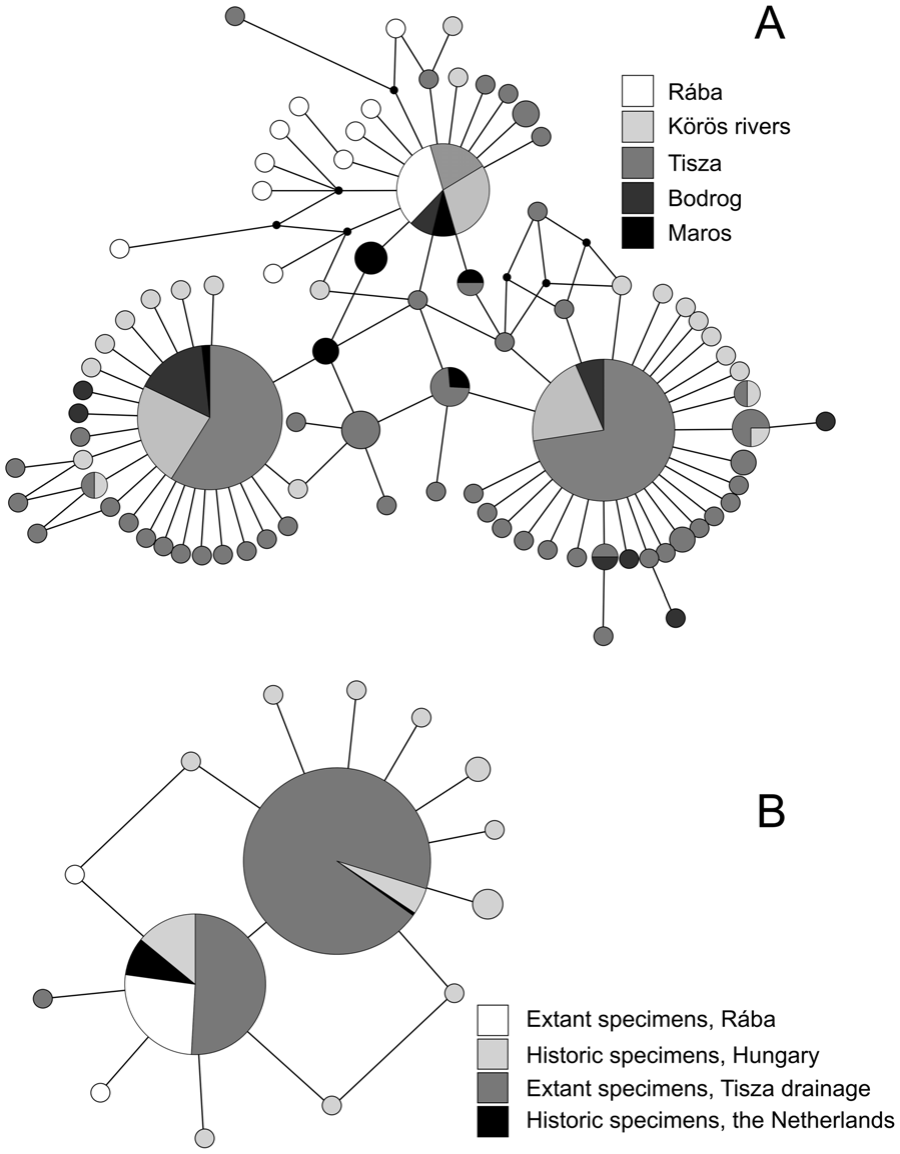
Relationships of *P. longicauda* haplotypes. A – median-joining networks of combined mtCOI and 16S haplotypes of successfully amplified extant specimens. B – median-joining networks of short mtCOI haplotypes of all extant and 24 historic specimens (Körös, Tisza, Maros and Bodrog marked as belonging to the Tisza catchment). Each circle represents a haloptype. The size of the circle indicates the frequency of the haplotype. Connecting lines represent single nucleotide substitutions.

We found high genetic diversity in the populations of both the Tisza and the Rába catchments. The 245 specimens comprised 87 haplotypes, most of them private to either the Tisza (76/77), or the Rába (9/10) catchments (Figure 3A). The overall haplotype diversity for the combined dataset was 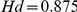, the nucleotide diversity was 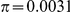. The mtCOI dataset contained 31 haplotypes (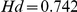, 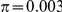). The 16S dataset contained 53 haplotypes (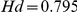, 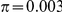). Most of the haplotypes were closely related and formed a triple star-like phylogeny with three widespread and numerous satellite haplotypes (Figure 3A). Many of the recorded haplotypes were found only in a single specimen. Three common haplotypes (separated by 3–4 mutation steps) contained 57% of all sequences. Two of these were recovered exclusively from the Tisza drainage, and the third was present in both the Rába and the Tisza systems.

Mitochondrial COI fragments of the specimens collected historically in the Netherlands and Hungary contained the same haplotypes as the extant specimens with the exception of a single individual, collected at an unknown location in Hungary (Figure 3B). The majority of specimens from the Netherlands shared a haplotype characteristic to both Rába and Tisza rivers, and one specimen showed the central Tisza haplotype. The two most common Tisza haplotypes collapsed into a single short mtCOI haplotype due to the reduced number of informative loci of the short mtCOI fragment.

**Figure 4:**
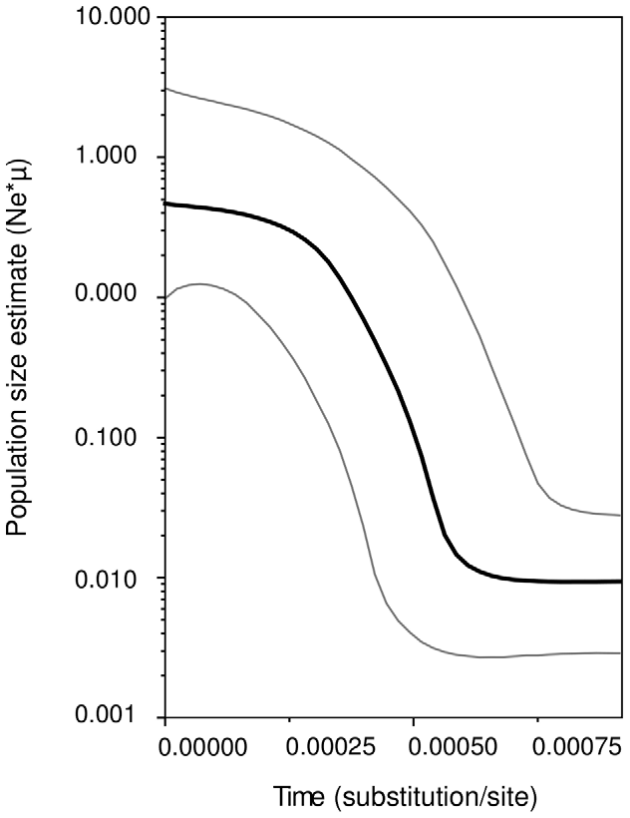
Demographic changes of *P. longicauda* populations. The black line shows the mean population size, estimated by Bayesian skyline plot. Grey lines show population sizes within the highest and lowest 95% probability density intervals.

Intense demographic changes of the Tisza and Rába populations were indicated by highly significant deviations from selective neutrality (Tajima's 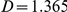, 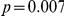; Fu's 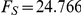, 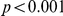). The Bayesian skyline plot (BSP) [27] also showed strong and rapid increase in the population size of about 2.5 orders of magnitude, followed by a plateau in demographic changes (Figure 4). The BSP suggested no recent population decline.

We found significant genetic differentiation between the Tisza (228 specimens) and Rába (17 specimens) (pairwise 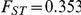, 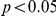). The exact test for population differentiation (ETPD) [28] also showed significant differentiation at 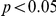 between the two river drainages. The pairwise comparison of populations showed that the single Rába population was significantly differentiated from every Tisza population. We observed only a few differences among the Tisza populations. The pairwise differentiation patterns were confirmed by the ETPD (see Table S3 for the results of pairwise comparisons). The analysis of molecular variance (AMOVA) of the Tisza populations revealed that most of the variance was found within populations (95.98%, 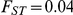, 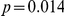). Relatively low variation was found among populations within the upper, middle and lower Tisza drainage population groups (2.92%, 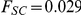, 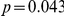). Variation was also low among these high-level structures (1.11%, 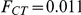, 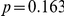). No correlation was found between the geographic and genetic distance matrices of the Tisza and Rába populations, after comparing them with a Mantel test (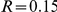, 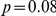).

The reduced, 196 bp-long mitochondrial sequence sets revealed significant differentiation between the historic Rhine and pooled Tisza samples (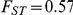, 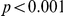). The Tisza and Rába samples were again differentiated (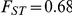, 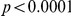). We found no differentiation between the historic Rhine specimens, and the Rába population (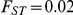, 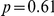). The Rhine and Rába samples were significantly differentiated from most Tisza populations in the pairwise comparison of all populations (Table S4). There were only few differences detected among the Tisza population pairs.

**Figure 5:**
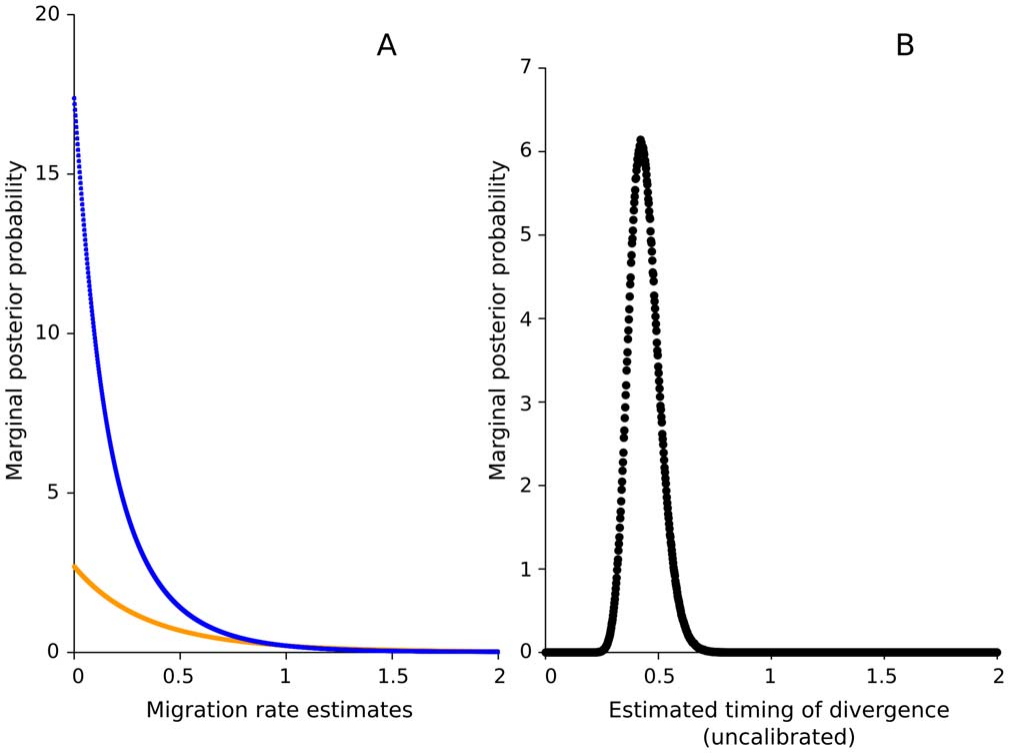
Likelihood of ongoing migrations and the timing of the disjunction between the Tisza and Rába catchments. A – The likelihood of ongoing migration to any directions is 0 (orange: migration from the Tisza to the Rába; blue: migration from the Rába to the Tisza). B – The complete separation of the Tisza and Rába populations most probably happened in the past. An extant connection between the two populations is unlikely.

The “Isolation with Migration” (IMa) [29] analysis confirmed that populations of the two extant ranges are separated in the present. The IMa suggested the highest likelihood for no extant migration between the Tisza and Rába populations (Figure 5A). Although the analysis is not time-calibrated, the IMa clearly indicated the complete divergence of the two populations in the present (Figure 5B). We also tested the likelihood of four migration scenarios between the Rába and the Tisza with MIGRATE-N [30]. These competing scenarios describe situations with no gene flow (1), symmetric gene flow (2), and two cases of unidirectional gene flow (3, 4) between the two drainages. As a result of model selection based on Akaike's information criterion [31], the scenario describing unidirectional migration from the Tisza toward the Rába received overwhelming support (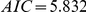, Akaike weight 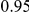). The symmetric migration scenario received low (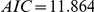, Akaike weight 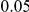) support. The no migration (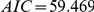, Akaike weight 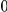), and the unidirectional Rába-Tisza migration scenarios (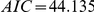, Akaike weight 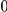) were both unsupported.

## Discussion

We found unexpectedly high haplotype diversity in the *P. longicauda* populations isolated in the Tisza and Rába catchments. In contrast, we found no genetic differences between the persisting Rába, and the extinct Rhine populations, despite the considerable geographic distance (1,000 km between the lower Rhine and the Rába), and the distinct river drainages. The recovered haplotype diversity (but not the nucleotide diversity) is comparable with that of other, still widespread species. Haplotype diversity was 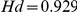 in the mayfly *Ameletus inopinatus* Eaton [32] (mtCOI data, 266 specimens, 620 bp, 42 haplotypes, 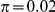). Haplotype diversity was 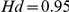 in a common caddisfly, *Drusus discolor* (Rambur) [14], [15], [33] (mtCOI data, 299 specimens, 498 bp, 69 haplotypes, 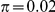). Both *A. inopinatus* and *D. discolor* have well diverged, geographically confined genetic lineages in European mountains.

Strongly reduced genetic diversity is frequently recorded for species which experienced extensive range losses [4], [5]. Contrary to the expectations, we found high haplotypic diversity (87 haplotypes in 245 investigated specimens) in the present-day range of *P. longicauda*. All but one of the haplotypes were private to either the Tisza or the Rába river systems. The geographic confinement of both major Tisza haplotypes, and all satellite haplotypes from both the Tisza and the Rába catchments suggest that the extant populations of these rivers had an independent history. This is supported also by the pairwise 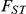 comparisons, the ETPD, Mantel test and IMa. The scenario selected by MIGRATE-N described unidirectional gene flow from the Tisza toward the Rába. Although the IMa and MIGRATE-N results may at first seem contradicting, the two methods answer different questions. IMa is suitable to detect recent divergences between two populations, whereas MIGRATE-N infers migration rates and returns the likelihood of migration scenarios among populations [34]. We thus interpret these results as evidence for historic, but not current gene flow between the Tisza and the Rába populations. Because all these populations belong to the same species, they must have been connected by gene flow in the past.

The next question that arises from these results is when did the Tisza and Rába populations diverge? Evidence for differentiation between the Tisza and Rába populations (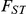, ETPD, IMa), unidirectional historical gene flow from Tisza to Rába, presence of relatively distant haplotypes private to each drainage, all suggest that this divergence happened before the last glacial maximum (LGM) and that there were two distinct glacial refugia for *P. longicauda*. The lower Danube drainage is considered as one of the important Pleistocene refugia of freshwater species [11]. Multiple Pleistocene refugia in the streams of the Danube drainage are also frequently reported [12]–[15], [18], [32], [35], [36]. The IMa showed that a very recent differentiation of the Rába and Tisza populations (mediated by human impact) is unlikely (Figure 5B), if we accept that 50–60 years since the species' extinction from the Danube were insufficient to accumulate enough genetic signal to result in this divergence. However, the zero-migration scenario between the Tisza and Rába ranges (which would clearly indicate separate refugia) received no support from the MIGRATE-N. The scenario describing symmetric radiation from a common refugia into the extant ranges also received very low support. The strongly supported model described unidirectional gene flow from the Tisza toward the Rába. In this regard, the persistence of *P. longicauda* during the LGM in only a single refugium (relatively close to the actual Tisza ranges) cannot be completely excluded. Assuming that mitochondrial mutation rates are not considerably higher in *P. longicauda* than in other insects, it is difficult to explain the presence of strongly diverged private haplotypes in the Rába (Figure 3) by an upstream colonization from a single lower-middle Danubian refugium, even if post-LGM colonizations upstream the Danube are among the well established paradigms of the European freshwater zoogeography [11]. We think that the best explanation for the observations is the LGM survival of the species in two middle-Danubian refugia, with a post-LGM introgression event from the lower-middle Danube drainage into populations upstream. *Palingenia longicauda* populations in the Rába can be considered independent from those in the Tisza drainage, and the presence of a shared haplotype is the result of the introgression event. The introgression was followed by a historic interruption of gene flow. Our results suggest that the Rába populations never went extinct, and their reappearance after 40 years of absence is the result of cryptic persistence (undetected by humans).

The 196 bp-long mitochondrial sequence set including the historic Rhine specimens did not contain sufficient numbers of informative loci to test hypotheses about migration patterns and disjunction events among populations in a coalescent framework. However, both 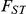-based comparisons and ETPDs confirmed the differentiation between the Tisza and Rába samples. The historic Rhine population was significantly differentiated from the Tisza samples, but not from the Rába. We think that two LGM refugia in the middle Danube drainage, followed by a post-LGM westward gene flow, and the subsequent colonization of the Rhine may best explain these patterns. Post-LGM colonization of the Rhine from the Danube was described for some other species [37], [38]. Our dataset has some limitations (strongly different sample sizes, short sequences with few informative loci), and we thus cannot completely exclude several alternative explanations of the results. We may have accidentally missed loci signaling for differentiation between the Rhine and Danube drainages. However, the studied fragment of the mitochondrial DNA is known for relatively high intraspecific variability [6], [14]. The fragment also proved sufficiently variable to recover the differences revealed by the full matrix of the Tisza and Rába. Colonization of the upper Danube drainage from independent Rhine and lower Danubian refugia would also explain the observed pattern. This would, however, contradict the long established theory of the post-LGM upstream colonization of freshwater organisms along the Danube [11]. As we are not aware of any evidence against this paradigm, we consider this possibility also unlikely. The lack of differentiation between the Rába and the Rhine suggests a relatively recent disjunction between these populations, although we could not calibrate a molecular clock. It is unlikely that gene flow between these populations existed until very recently. The actually existing barriers between the two drainages would efficiently prevent this. The reactivation of the European rivers after the LGM had an immense impact on the hydrology of the continent [39], and these events seem more suitable to facilitate contact between major river systems.

Probably the most surprising result of our work is the observed high genetic diversity in the present ranges. This is likely the result of a fortunate overlap between two of the species' last glacial maximum refugia, and its current ranges. The middle-lower Danube catchment served as an important LGM refugium for many other European stream and wetland species, e.g. fishes, caddisflies, newts, etc. [11]–[14], [40]. Our results thus support the importance of the middle-lower Danube drainage in the conservation of the European freshwater genetic resources. This region is known to play a similar role for other, biogeographically Danubian taxa sensu B
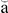
n
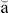
rescu [11], such as the Danube Salmon (*Hucho hucho*) (Berne Convention, Appendix III, IUCN endangered) [24], [41], the Carpathian Brook Lamprey (*Eudontomyzon danfordi*), and the Danube Gudgeon (*Romanogobio uranoscopus*) (Berne Convention, Appendix III, IUCN endangered) [24].

In a striking contrast with the high genetic diversity of the presently isolated ranges, all historic *P. longicauda* specimens from the Rhine catchment had haplotypes present today in the Rába and the Tisza systems (Figure 3B). This is surprising, especially when considering the large geographic distances between the lower Rhine and the Tisza/Rába. We expected strong genetic differentiation between the Danube and Rhine drainages, given that strong differentiation, and even cryptic taxa of European freshwater species are frequently discovered even in geographically close ranges, e.g. [13]–[16], [18], [32], [36], [42]–[45]. The star-like shape of the haplotype network [45]–[48], the highly significant deviations from selective neutrality, and the BSP estimates (Figure 4) all suggest an important and relatively recent increase in effective population size. The inferred increase is presumably also responsible for the partial formation of the extant genetic diversity, particularly the high numbers of satellite haplotypes diverged by a single mutation step from one of the central haplotypes. Given the limitations of the museum dataset (relatively few specimens and short nucleotide sequences), we can only speculate about source areas of the Rhine colonization: these were probably located in the middle Danube drainage. This supports the view that the ongoing reintroduction efforts of *P. longicauda* into the Rhine catchment [25] use genetically adequate source materials.

An overlap between former LGM refugia and areas less impacted by recent human activities may account for the preservation of significant intraspecific genetic diversity. The complete extinction of species from large areas may not significantly impact their diversity if the extinction affects only recently colonized areas. On the other hand, even small-scale range reductions may strongly impact intraspecific diversity, if they are affecting former (e.g. glacial) refugia.

The long-term cryptic persistence of *P. longicauda* in the Rába suggests that small populations may still survive on the formerly vast distribution range. Recent observations in distant areas (e.g. the Danube Delta) [49] may also be attributed to cryptic survival instead of recent range expansion. This emphasizes the potential of false absences in the conservation of this formerly widespread species, and the importance of extensive surveillance efforts accounting for the opportunities of the “unknown unknowns” [50]. We suggest that the re-discovery and active local protection of persisting cryptic populations of *P. longicauda* may be just as important for their long-term conservation, as active reintroductions [25], and the targeted monitoring of the Tisza and Rába populations [26].

## Materials and Methods

### Focal species

*Palingenia longicauda* is well-known since ancient times due to its size and brief, highly synchronized mass swarming. The first written record that likely refers to this species originates from Aristotle in the 4th century B.C. from Greece [20]. The first scientific treatment was given by Clutius in 1635 from Belgium [20]. The species inhabited the probably most vulnerable European freshwaters: lowland navigable rivers. The eroding banks of these rivers have often been reinforced by riprap or concrete, a practice that destroyed the habitats of the larvae. The larvae develop for 3 years almost exclusively in the continuously eroding steep outer riverbanks composed mostly of clay [51], [52]. Mass emergence as subimagos and imagos after the long larval development lasts only for a few hours. Biogeographically, *P. longicauda* is considered an expansive pontic element, which was present in almost every large European river [49]. After its extinction along the lower Danube in 1974 (including the Danube Delta) [19], the Tisza river in eastern Hungary was considered the last known range where mass swarms still occur regularly. A small population was discovered in the Rába river in Hungary [21] after about 40 years of absence of reports. The Rába river is now considered the westernmost edge of the present distribution [52]. The recent finding of a few specimens in the Danube Delta is attributed to ongoing range expansion [49].

### Field and laboratory methods

We obtained 244 larvae from well-documented *P. longicauda* populations of the Tisza drainage and 18 larvae from the rediscovered habitat on the Rába river (Figure 2, Table S1). We collected larvae along the riverbank with a “báger”, a conical metal cylinder with an opening of 25 cm, historically used by fishermen for the same purpose. We sampled across all suitable habitats (eroding banks) at each locality. The specimens were preserved in 96% ethanol until DNA extraction, and they were deposited in the Senckenberg Museum (Frankfurt am Main) for long-term storage. We also used one or two legs of 37 historic dried museum specimens from the following collections: Natural History Museum (London), Senckenberg Museum (Frankfurt am Main), Natural History Museum (Budapest), and Natural History Museum (Vienna) (for more details see Table S2). There was no information available about the sampling methods of the museum specimens. All successfully amplified museum specimens were imagos/subimagos, thus they were likely collected during the synchronized mass swarms.

We extracted DNA using the DNeasy Blood & Tissue Kit for the extant specimens, and the QIAamp DNA Investigator Kit for the museum specimens (both Qiagen, Hilden, Germany), according to the manufacturers protocols. Legs of the dried museum individuals were homogenized in a Qiagen TissueLyser II. We extracted DNA from museum specimens in a laboratory dedicated to the pre-PCR processing of non-invasively collected and historic specimens. The laboratory is physically strictly separated from other DNA-laboratories in order to prevent any contamination from samples with high DNA content. Standard routines for the processing of historic samples were considered, such as strict rules for laboratory access, use of filter tips, and regular decontamination of equipment. In addition, negative controls were included in all extraction and amplification steps as contamination check. We amplified an approximately 600 bp long fragment of the mtCOI gene with the primers Jerry [53] and S20 [14], and an approximately 520 bp-long fragment of the 16S ribosomal rRNA with primers 16Sar [53] and 16SB2 [54]. We successfully and repeatedly amplified a short, 196 bp long mtCOI fragment of the museum specimens with newly designed primers (PalJS20Int-1F: 5′TGATTATTGCCGTTCCTACTGG; PalJS20Int-1R: 5′AATGAAAATGGGCTACTACG). We set up PCR reactions on museum specimens in a laboratory dedicated exclusively for the pre-PCR treatment of non-invasively collected or ancient mammalian material. Amplicons were sequenced on an ABI 3730 DNA Analyzer (Applied Biosystems). We manually edited the tracefiles and aligned sequences in BioEdit [55]. After creating the alignment, we compared the sequences and re-checked the tracefiles whenever a mutation was found only in one or two specimens.

### Statistical analyses of population structure

We ensured that the mtCOI and 16S datasets do not contain conflicting phylogenetic information by visually inspecting the topology of two bootstrapped (10,000 replicates) neighbor-joining trees in SeaView v. 4 [56]. We computed haplotype diversity (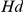) and nucleotide diversity (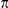) for the individual mtCOI and 16S, and also for the concatenated dataset in DnaSP v. 5 [57]. We used median-joining networks [58] implemented in Network 4.5.1.6 (Fluxus Technology) to visualize relationships among haplotypes. We calculated 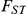 values on the basis of pairwise differences, and ran an ETPD [28] to estimate differentiation between the Rába population and the pooled populations of the Tisza. We also calculated 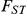 values and the ETPD for all individual population pairs from the Tisza and Rába. Similarly, we calculated pairwise 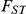 values and performed an ETPD with the 196 bp mtCOI sequence set. First we compared the historic Rhine, the Rába and pooled Tisza populations. Second, we made pairwise comparisons among all individual populations. We performed 10,000 permutations to estimate the statistical significance of pairwise 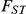 values. The length of the Markov chain was 100,000 steps, with 10,000 steps for initial de-memorization (burn-in) in the case of the ETPDs. We used Tajima's 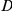 and Fu's 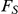 tests for selective neutrality to search for signals of demographic changes. We analysed the molecular variance of Tisza populations. The AMOVA was run with 10,000 permutations, with a distance matrix computed on the basis of pairwise differences. Individual Tisza populations were treated as low-level structures. Individual populations were grouped into high-level structures according to the position of these populations along the Tisza drainage basin: upper Tisza (Aranyosapáti, Cigánd, Gulács, Szegi, Tarpa, Tiszatardos, Zsurk), middle Tisza (Körösladány, Poroszló, Gyula, Szeghalom), lower Tisza (Algyö, Csongrád, Ferencszállás). We also checked if the geographic distance of Tisza and Rába populations may have played a role in the formation of the genetic structures observed there, correlating the 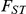 and geographic distance matrices with a Mantel test. Geographic distance among populations was measured along the rivers in QGIS [59]. We calculated pairwise 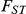 values, the ETPD, Tajima's 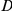, Fu's 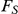, and run the Mantel test and AMOVA in Arlequin 3.11 [60].

We reconstructed changes in effective population sizes using a Bayesian skyline plot [27] in BEAST 1.5.4 [61]. We selected the GTR+I+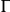 as substitution model with Akaike's information criterion in Modeltest 3.7 [62]. We used a strict molecular clock model, with the timing of events estimated in numbers of substitutions/sites, as no calibration dates were available for the analysis. We used a UPGMA-generated starting tree in our piecewise-constant Bayesian skyline model, with 10 groups. All priors for model parameters and statistics had default distributions. We ran 12 tests in parallel for 50 million generations each, and sampled them for every 5000 generations. We combined the results in LogCombiner 1.5.4 (part of the BEAST program). The convergence of the tests was assessed in Tracer 1.5 [63] after discarding 10% of the samples as burn-in.

We estimated the divergence, lineage sorting, ongoing gene flow and the likelihood of migration of the Tisza (population 1) and the Rába (population 2) in IMa [29] and MIGRATE-N v. 3.2.6 [30]. We could not compare the extinct Rhine and extant populations with coalescent methods, as there were insufficient informative sites in the short (196 bp long) mtCOI dataset for coalescent inferences. We ensured the convergence of the IMa results by 5 exploratory runs. We used a single value (10) for all priors for the first exploratory run, then we refined the prior maximum values after each run, until the posterior estimates were fully contained within the bounds of the prior distribution. We established 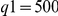, 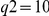, 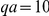, 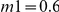, 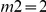, 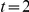 as prior maximum values for the final test, according to the outputs of the 5 preliminary runs. In MIGRATE-N we conducted an exploratory run to find suitable starting 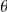 values for both populations, generating the initial 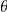 and migrant values from 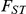 calculations. We also checked the convergence of the results by running 3 trial tests with strongly different starting 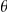 values for both populations (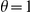, 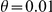, 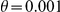). We set up four migration scenarios between the Tisza and Rába. The first scenario assumed no migration between the two populations. The second scenario assumed symmetric migration. The third scenario described a unidirectional migration from the Tisza to the Rába. The fourth scenario was a unidirectional migration from the Rába to the Tisza. We tested their likelihoods against an unconstrained migration model with the likelihood-ratio-test option of MIGRATE-N. We employed a genealogy search with four chains heated under a static heating scheme (temperatures 1, 2, 4, 8). We ran 10 short chains for 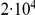 generations, and 3 long chains for 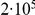 generations. We recorded 1000 trees at regular intervals from both short and long chains. We discarded the first 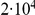 trees as burn-in. We used the likelihood-estimates and the number of free parameters to calculate the Akaike information criterion [31] and the Akaike weight of each scenario.

## Supporting Information

Table S1 **Collection data of extant specimens.** Abbreviations: HNP – Hortobágy National Park; KMNP – Körös-Maros National Park; KNP – Kunsági National Park; BUNP – Balaton Uplands National Park; KM – Kristóf Málnás; BAP – BioAqua Pro Ltd.
(DOC)

Table S2 **Collection data.** Historically collected P. longicauda specimens processed for amplifying a short mtCOI fragment (MTM – Magyar Természettudományi Múzeum [Hungarian Natural History Museum], Budapest; SNM – Senckenberg Naturmuseum, Frankfurt am Main; NMW – Naturhistorisches Museum Wien; NHM – Natural History Museum, London).
(DOC)

Table S3
**Pairwise** 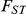 **values (lower left) and significant ETPD (upper right) results of extant populations.** The tests are calculated with combined mtCOI and 16S sequences (936 bp). 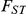 values in bold are significant at 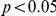. “+” ETPD values mark significant population differentiation at 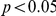.(XLS)

Table S4
**Pairwise** 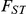 **values (lower left) and significant ETPD (upper right) results of extant and museum (Rhine) populations.** The tests are calculated with combined the short mtCOI sequences (196 bp). 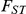 values in bold are significant at 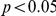. “+” ETPD values mark significant population differentiation at 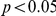.(XLS)
