# Supplementary material for: Species History Masks the Effects of Human-Induced Range Loss – Unexpected Genetic Diversity in the Endangered Giant Mayfly Palingenia longicauda
Source: PLoS One. 2012 Mar 8;7(3):e31872. doi: 10.1371/journal.pone.0031872 (PMC3297596; doi:10.1371/journal.pone.0031872)
Supplement: Table S1 — Collection data of extant specimens. Abbreviations: HNP – Hortobágy National Park; KMNP – Körös-Maros National Park; KNP – Kunsági National Park; BUNP – Balaton Uplands National Park; KM – Kristóf Málnás; BAP – BioAqua Pro Ltd. (DOC) [file pone.0031872.s001.doc]

Species’ history masks the effects of human-induced range loss – unexpected genetic diversity in the endangered giant mayfly *Palingenia longicauda*

Miklós Bálint, Kristóf Málnás, Carsten Nowak, Jutta Geismar, Éva Váncsa, László Polyák, Szabolcs

Lengyel, Peter Haase

Supporting table S1. Collection data of extant specimens. Abbreviations: HNP – Hortobágy National Park; KMNP – Körös-Maros National Park; KNP – Kunsági National Park; BUNP – Balaton Uplands National Park; KM – Kristóf Málnás; BAP – BioAqua Pro Ltd.

|  | **Locality** | **River name** | **N** | **E** | **Specimens** | **Authorization number, issuing authority and authorization holder** |
| --- | --- | --- | --- | --- | --- | --- |
| 1 | Tarpa | Tisza | 48.104° | 22.527° | 21 | 9544/2007, HNP, KM |
| 2 | Gulács | Tisza | 48.068° | 22.477° | 16 | 9544/2007, HNP, KM |
| 3 | Aranyosapáti | Tisza | 48.220° | 22.274° | 18 | 12558-2/2008, HNP, KM |
| 4 | Tiszatardos | Tisza | 48.377° | 22.233° | 16 | 12558-2/2008, HNP, KM |
| 5 | Zsurk | Tisza | 48.411° | 22.219° | 16 | 12558-2/2008, HNP, KM |
| 6 | Cigánd | Tisza | 48.249° | 21.924° | 18 | 12558-2/2008, HNP, KM |
| 7 | Szegi | Bodrog | 48.216° | 21.411° | 10 | 12558-2/2008, HNP, KM |
| 8 | Szegi | Bodrog | 48.195° | 21.376° | 9 | 12558-2/2008, HNP, KM |
| 9 | Poroszló | Tisza | 47.640° | 20.641° | 19 | 12558-2/2008, HNP, KM |
| 10 | Szeghalom | Sebes-Körös | 46.986° | 21.174° | 18 | 754/2008, KMNP, KM |
| 11 | Körösladány | Kettős-Körös | 46.935° | 21.043° | 28 | 754/2008, KMNP, KM |
| 12 | Gyula | Fekete-Körös | 46.704° | 21.304° | 8 | 754/2008, KMNP, KM |
| 13 | Csongrád | Tisza | 46.351° | 20.206° | 18 | 588-2/2008, KNP, KM |
| 14 | Algyő | Tisza | 46.351° | 20.200° | 10 | 754/2008, KMNP, KM |
| 15 | Ferencszállás | Maros | 46.223° | 20.374° | 15 | 754/2008, KMNP, KM |
| 16 | Rum | Rába | 47.113° | 16.837° | 18 | 1222-2/2008, BUNP, BAP |
