# Supplementary material for: Species History Masks the Effects of Human-Induced Range Loss – Unexpected Genetic Diversity in the Endangered Giant Mayfly Palingenia longicauda
Source: PLoS One. 2012 Mar 8;7(3):e31872. doi: 10.1371/journal.pone.0031872 (PMC3297596; doi:10.1371/journal.pone.0031872)
Supplement: Table S2 — Collection data. Historically collected P. longicauda specimens processed for amplifying a short mtCOI fragment (MTM – Magyar Természettudományi Múzeum [Hungarian Natural History Museum], Budapest; SNM – Senckenberg Naturmuseum, Frankfurt am Main; NMW – Naturhistorisches Museum Wien; NHM – Natural History Museum, London). (DOC) [file pone.0031872.s002.doc]

Species’ history masks the effects of human-induced range loss – unexpected genetic diversity in the endangered giant mayfly *Palingenia longicauda*

Miklós Bálint, Kristóf Málnás, Carsten Nowak, Jutta Geismar, Éva Váncsa, László Polyák, Szabolcs

Lengyel, Peter Haase

Supporting table S2. Collection data of historically collected *P. longicauda* specimens processed for amplifying a short mtCOI fragment (MTM – Magyar Természettudományi Múzeum [Hungarian Natural History Museum], Budapest; SNM – Senckenberg Naturmuseum, Frankfurt am Main; NMW – Naturhistorisches Museum Wien; NHM – Natural History Museum, London).

| **ID** | **Locality** | **River** | **Label** | **Collector** | **Collection date** | **Deposited** | **Successful PCR** |
| --- | --- | --- | --- | --- | --- | --- | --- |
| HM263 | Szeged | Maros |  | Csoknya | 9. June 1971 | MTM | X |
| HM265 | Szeged | Maros |  | Csoknya | 9. June 1971 | MTM |  |
| HM266 | Szeged | Maros |  | Csoknya | 9. June 1971 | MTM | X |
| HM267 | Szeged | Maros |  | Csoknya | 9. June 1971 | MTM | X |
| HM268 | Szeged | Maros |  | Csoknya | 9. June 1971 | MTM | X |
| HM269 | Szeged | Maros |  | Csoknya | 9. June 1971 | MTM |  |
| HM270 | Szeged | Tisza |  | Wirth J | 12. June 1967 | MTM | X |
| HM271 | Szeged | Tisza |  | Wirth J | 12. June 1967 | MTM | X |
| HM272 | Szeged | Tisza |  | Wirth J | 12. June 1967 | MTM | X |
| HM273 | Szeged | Tisza |  | Wirth J | 12. June 1967 | MTM | X |
| HM288 | Szeged | Tisza |  | Wirth J | 12. June 1967 | MTM | X |
| HM292 | Szeged | Tisza |  | Wirth J | 12. June 1967 | MTM | X |
| HM293 | Szeged | Tisza |  | Wirth J | 12. June 1967 | MTM | X |
| HM294 | Szeged | Tisza |  | Wirth J | 12. June 1967 | MTM | X |
| HM295 | Szeged | Tisza |  | Wirth J | 12. June 1967 | MTM | X |
| HM296 | Vasegerszeg | Rába |  | Wirth J | 22. June 1962 | MTM | X |
| HU303 | Hungary | Danube | Hungaria, Scigetcsep, Cserva |  |  | SNM |  |
| HU304 | Hungary |  | Hungaria, Standinger |  |  | SNM | X |
| XU305 | Xupanje | Sava |  | Heyden |  | SNM |  |
| HU306 | Hungary | Danube | Hungaria, Scigetcsep, Cserva |  |  | SNM |  |
| XU307 | Xupanje | Sava |  | Heyden |  | SNM |  |
| XU308 | Xupanje | Sava |  | Heyden |  | SNM |  |
| LI309 | Hamm | Lippe | Palingenia longicauda (Oliv.) aus der Lippe bei Hamm in Westphalen. Cornelius v. Heyden | Cornelius v. Heyden |  | SNM |  |
| LI310 | Hamm | Lippe | Palingenia longicauda (Oliv.) flosaque. Illig. Swammerdamiana Ltr. Aus der Lippe bei Ham in Wesphalen. Cornelius | Cornelius |  | SNM |  |
| LI311 | Hamm | Lippe | Palingenia longicauda (Oliv.) wahrend der Häutung Hamm. Cornelius | Cornelius |  | SNM |  |
| WA312 | Hamm | Lippe | Palingenia longicauda (Oliv.) Wahrender Hautung Hamm. Cornelius | Cornelius |  | SNM |  |
| HA313 | Hamm | Lippe | P. longicauda Puppe (Oliv.) Hamm. Cornelius | Cornelius |  | SNM |  |
| WE315 | Westfalen |  | Palingenia longicauda Kolenati F.Westphalien li Vorderbein | Kolenati |  | NMW |  |
| NL316 | The Netherlands |  |  |  |  | NHM | X |
| NL317 | The Netherlands |  |  |  |  | NHM | X |
| NL318 | The Netherlands |  |  |  |  | NHM | X |
| NL319 | The Netherlands |  |  |  |  | NHM | X |
| NL320 | The Netherlands |  |  |  |  | NHM | X |
| NL322 | The Netherlands |  |  |  |  | NHM | X |
| HU323 | Hungary |  |  |  |  | NHM | X |
| HU324 | Hungary |  |  |  |  | NHM | X |
| HU325 | Hungary |  |  |  |  | NHM | X |
